# Using Micromachined Molds, Partial-curing PDMS Bonding Technique, and Multiple Casting to Create Hybrid Microfluidic Chip for Microlens Array

**DOI:** 10.3390/mi10090572

**Published:** 2019-08-29

**Authors:** Pin-Chuan Chen, Ren-Hao Zhang, Liang-Ta Chen

**Affiliations:** Department of Mechanical Engineering, National Taiwan University of Science and Technology, Taipei 10607, Taiwan

**Keywords:** microlens array, microfluidics, micromilled microfluidic devices

## Abstract

In a previous study, we presented a novel manufacturing process for the creation of 6 × 6 and 8 × 8 microlens arrays (MLAs) comprising lenses with diameters of 1000 μm, 500 μm, and 200 μm within an area that covers 10 mm × 10 mm. In the current study, we revised the manufacturing process to allow for the fabrication of MLAs of far higher density (15 × 15 and 29 × 29 within the same area). In this paper, we detail the revised manufacturing scheme, including the micromachining of molds, the partial-curing polydimethylsiloxane (PDMS) bonding used to fuse the glass substrate and PDMS, and the multi-step casting process. The primary challenges that are involved in creating MLAs of this density were ensuring uniform membrane thickness and preventing leakage between the PDMS and glass substrate. The experiment results demonstrated that the revised fabrication process is capable of producing high density arrays: Design I produced 15 × 15 MLAs with lens diameter of 0.5 mm and fill factor of 47.94%, while Design II produced 29 × 29 MLAs with lens diameter of 0.25 mm and fill factor of 40.87%. The partial-curing PDMS bonding system also proved to be effective in fusing PDMS with glass (maximum bonding strength of approximately six bars). Finally, the redesigned mold was used to create PDMS membranes of high thickness uniformity (coefficient of variance <0.07) and microlenses of high lens height uniformity (coefficient of variance <0.15).

## 1. Introduction

Optics and optoelectronics are playing increasingly important roles in the fields of communication, processing, sensing, and display systems. Micro-optical devices are critical components in many electronic devices that require miniaturized photonic systems with improved integration, precision, multifunctionality, and energy savings [1]. Refractive microlens arrays (MLAs) refract incident light in accordance with Snell’s law. The primary objective in the implementation of MLAs is to increase the illumination and efficiency of optical systems. These devices are widely used in optical fiber coupling [2], high-efficiency charge coupled devices (CCDs) [3], light diffusers [4], liquid crystal displays (LCDs) [5], wave-front sensors [6], and image recorders [7]. MLAs are also widely used in the field of biomedicine. Di et al. [8] used photolithography and thermal reflow to fabricate a multi-spectral 9 × 9 lens array that aimed at assisting individuals with color blindness. Each lens was coated with a filter of a different color to simultaneously capture light in a range of wavebands. The efficacy of the device was verified in experiments using color blindness testing cards. This kind of compact multi-spectral lens is also suited to biomedical applications. 

MLAs can be fabricated using glass, polymers, and semiconductors. Glass provides a high transition temperature, excellent chemical stability [9], and excellent mechanical properties, which means that it can be used in high-temperature and high-humidity environments [10]. MLAs that were fabricated on a silicon substrate are used primarily for infrared devices due to the fact that silicon is transparent at wavelengths between 1.3~1.6 μm [11]. Polymers are also used for optical devices due to their good mechanical characteristics, low cost, and ease of mass production [12]. The thermoplastics most commonly used in optical components include polycarbonate (PC), polystyrene (PS), and poly (methyl methacrylate) (PMMA) [13]. 

Many research groups have developed methods for the fabrication of MLAs, such as photolithography [14,15,16], thermal reflow [17,18], laser [19,20], argon ion beam etching [21,22], inkjet printing [23], and hot embossing [24,25]. Lin et al. [26] combined a thermal flow process with repeating spin coating to create hexagonal MLAs with a fill factor of 100%. Chronis et al. [27] fabricated an elastomer-based liquid-filled tunable MLA while using soft lithography. The focal length of the micro lenses is pneumatically controlled via a microfluidic network to ensure even pressurization of the micro lenses to create a device with a focal length that ranged from hundreds of microns to several millimeters. Hongwen et al. [28] used PDMS elastic material as a membrane with rubber to seal the liquid-filled array. Pressure that was provided by an external servo motor altered the shape of the upper PDMS film to create a lens. Hsiharng et al. [29] created two masks while using double exposure photolithography to create a lens and barricade capable of providing precise control over a variable-focus lens. The entire device was fabricated using PDMS to decrease the incompatibility between different materials. Yu et al. [30] reported a tunable double-focus micro lens that was created while using single-step SU-8 photolithography. The thickness of the forming layer determined the difference in thickness between the central and peripheral regions of the membrane. 

In a previous study, we demonstrated an economical method by which to create spherical MLAs through the micro milling and casting of PDMS in conjunction with ultraviolet (UV) curable adhesive [31]. In that process, micro milling was used to fabricate the first mold on a PMMA. PDMS casting and UV adhesive casting were then sequentially used to create PDMS MLAs. This fabrication process is based on the adjustment of pressure within the micro channel to deform the top PDMS membrane in order to produce a well-defined MLA, in terms of lens height, curvature, and focal length. In that study, we succeeded in creating 6 × 6 and 8 × 8 MLAs within a square area of 10 mm × 10mm, and the experiment results clearly demonstrated the advantage of this proposed method, which was able to create a high lens height/diameter ratio MLAs (for example, a 500 μm diameter MLAs fabricated from our method could have a lens height of 250 μm, while a commercial 500 μm diameter MLAs could only have a lens height of 100 μm). A denser MLA would be required for most practical applications, thus, a denser MLA would be required for most practical applications. Thus, our objective in the current research was to create 15 × 15 and 29 × 29 MLAs within an area of 10 mm × 10 mm on PDMS substrates. In preliminary experiments, leakage between the PDMS and glass substrates was a critical issue. Considerable effort was also required to create PDMS membranes of uniform thickness. 

In this study, we intentionally redesigned the molds for casting the uniform PDMS membrane and adopted a partial-curing PDMS bonding technique to increase the bonding strength, and they both approached executed herein helped achieve a denser MLAs. Several experiments were conducted to characterize the efficacy of the proposed fabrication process in terms of uniformity in membrane thickness, bonding strength between the PDMS and glass substrate, and the lens height of the final PDMS MLAs.

## 2. Revised Microfabrication Process

### 2.1. Revision from Previous Microfabrication Process 

The previous and current fabrication processes are both based on the deformation of a polydimethylsiloxane (PDMS) membrane to form spherical microlenses on a microfluidic chip. Following the fabrication of PMMA molds, a multiple casting method (PDMS and UV adhesive casting) is used to create the final PDMS MLAs. In the current study, we revised two of the previous steps to fabricate MLAs of higher density. We also redesigned the first mold and used partial-curing PDMS bonding to fuse the PDMS and glass substrate. 

**Figure 1:**
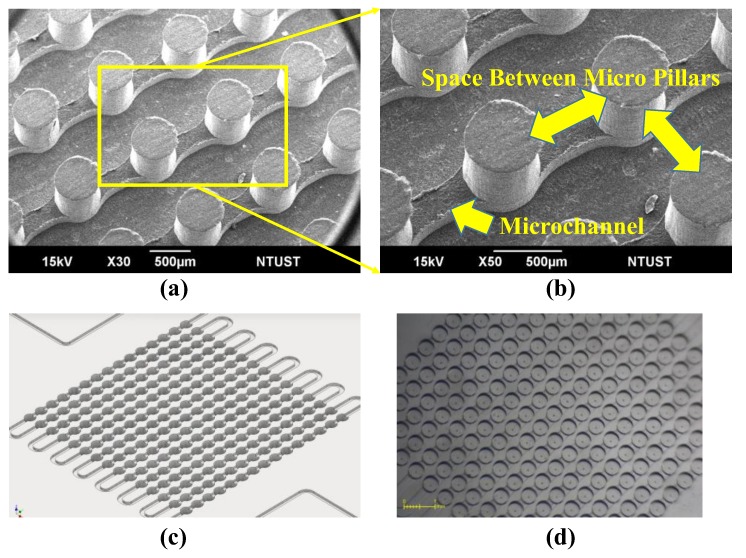
(**a**) SEM image showing standing micro pillars on poly (methyl methacrylate) (PMMA) substrate from our previous study; (**b**) SEM image showing how space between micromilled pillars limited density of microlens arrays (MLAs), the height of the micro pillar is 0.36 mm while the microchannel has 0.4 mm height and 0.4 mm width; (**c**) micro-cavities were drilled into PMMA substrate as an alternative to micro-pillars; and, (**d**) top-view image of micro-cavities drilled into PMMA substrate.

In the previous microfabrication process, micromilling was used to create standing micro pillars on the PMMA substrate, as shown in Figure 1a. The arrows in Figure 1b indicate the space between the micro pillars, which obviously must exceed the diameter of the micromilling bit. In the revised microfabrication process, we drilled cavities in the PMMA substrate (instead of milling micro-pillars) to create the first mold (see Figure 1c). Essentially, this fabrication process made it possible to increase the density of the MLA by overcoming the space limitation between the pillars; i.e., the cavities were not limited by the diameter of the micromilling bit. Figure 1d presents a top-view image showing the cavities that were drilled into the PMMA substrate.

In the previous fabrication process, plasma bonding was used to attach the PDMS membrane to the glass substrate; however, in preliminary experiments, the bonding strength was insufficient for preventing leakage or debonding during the UV adhesive casting process, particularly when the density of the MLAs was increased. In the current study, we opted for partial-curing PDMS bonding to provide heterogeneous bonding between the PDMS and glass substrate. The partial-curing PDMS bonding scheme included the following steps: (1) The glass substrate was first cleaned using isopropyl alcohol (IPA) and water. (2) A 10:1 PDMS mixture was poured onto the glass slide, followed by spin-coating at 1500 rpm for 40 s to create a PDMS layer with thickness of 15 μm. (3) The PDMS-coated glass slide was then placed in an 80 °C oven for two minutes to perform partial curing. (4) The solid PDMS membrane was brought into contact with the glass slide with the partially-cured PDMS coating. (5) The PDMS membrane and the glass slide were placed in the oven at 80 °C for 20 minutes to complete the curing process.

### 2.2. Design of MLAs

**Figure 2:**
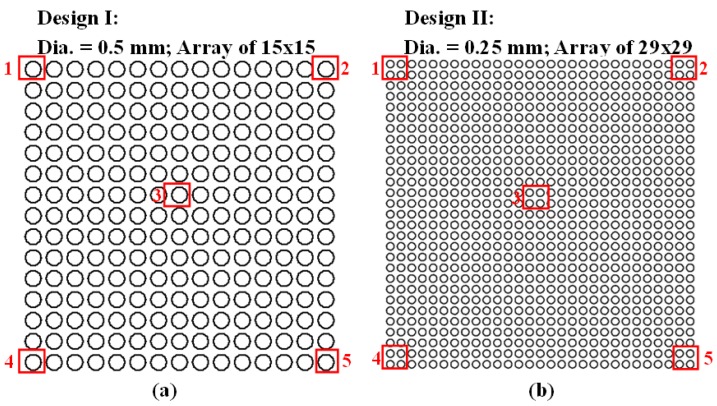
Proposed MLA designs: (**a**) Design I: 15 × 15 array comprising microlenses with 0.5 mm diameter and 0.15 mm spacing; and, (**b**) Design II: 29 × 29 array comprising microlenses with 0.25 mm diameter and 0.1 mm spacing. Red squares indicate the five locations from which measurements were obtained to determine membrane thickness uniformity and lens height uniformity of samples.

In this study, we designed and fabricated two MLAs. As shown in Figure 2a, Design I was a 15 × 15 MLA that comprised 225 microlenses with diameter of 0.5 mm and spacing of 0.15 mm. As shown in Figure 2b, Design II was a 29 × 29 MLA comprising 841 microlenses with a diameter of 0.25 mm and spacing of 0.1 mm. The MLAs in both designs comprised spherical microlenses within an area covering 10 mm × 10 mm.

Fill factor is a measure used to estimate the density of MLAs, as follows:

(1) $$\mathrm{Fill}\;\mathrm{Factor}\left(\%\right)=\frac{\pi r^{2}N}{Overall\;Area}\;\times \;100\%$$

where *r* is the radius of a single MLA and *N* is the number of MLAs. Based on this equation, the fill factor of Design I is 47.9% and the fill factor of Design II is 40.8%.

### 2.3. Microfabrication Process

**Figure 3:**
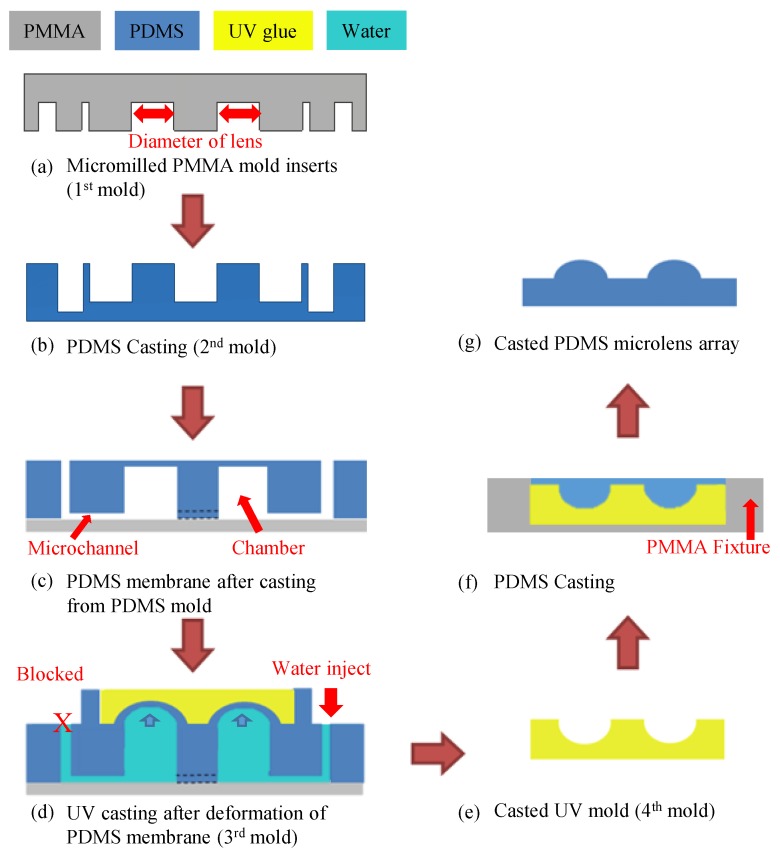
Overall revised fabrication process: (**a**) micromilling of mold; (**b**) casting of polydimethylsiloxane (PDMS) mold from micromilled mold; (**c**) casting of PDMS membrane from assembled mold, the assembled mold is described in Section 2.3.3, then using partial-curing PDMS bonding to form hybrid microfluidic chip for fabrication of MLAs; (**d**) after water was injected into the microchannel and the outlet was blocked, UV adhesive casting on hybrid microfluidic chip for fabrication of UV adhesive mold; (**e**) demolding of UV adhesive mold; (**f**) assembly of UV molds with PMMA fixture to control thickness of final PDMS MLAs; and, (**g**) completed PDMS MLA.

Figure 3 illustrates the revised fabrication process that was used to create MLAs of higher density, and Supplementary Material Figure S1 shows the three-dimensional (3D) fabrication process. Figure 3a shows the micromilled molds that were used in the fabrication of the PDMS mold in Figure 3b. Subsequently, PDMS mold was assembled with other two PMMA molds, which was used to form the PDMS membrane. The PDMS membrane was then bonded to a glass substrate via partial-curing PDMS bonding to form a hybrid microfluidic chip, as shown in Figure 3c. Water that was injected into the microchannel of the glass-PDMS hybrid microfluidic chip under pressure caused the elastic PDMS membrane to deform into a spherical shape. As shown in Figure 3d, the deformed PDMS membrane on the hybrid microfluidic chip was then used as a mold for UV adhesive casting, wherein UV adhesive was poured over the membrane and then quickly hardened under exposure to UV radiation. The resulting component was then used as a mold in subsequent PDMS casting (Figure 3e). Assembling UV molds with PMMA fixture was to control the thickness of final PDMS MLAs. In the final step, PDMS casting was again used to transfer the spherical concave shape from the UV mold to the PDMS substrate (Figure 3f). The resulting PDMS MLAs (Figure 3g) were then examined to determine the uniformity in height. In the revised method, the diameter of the MLAs was derived from the diameter of the drilled micro-cavities (Figure 3a). The thickness of the PDMS membrane was derived from the mold design (Figure 3b). The pressure within the microchannel was used to control the degree to which the PDMS membrane deformed, as well as the physical dimensions of the final MLAs (Figure 3d). 

#### 2.3.1. First Polymeric Mold with Micromilling 

**Figure 4:**
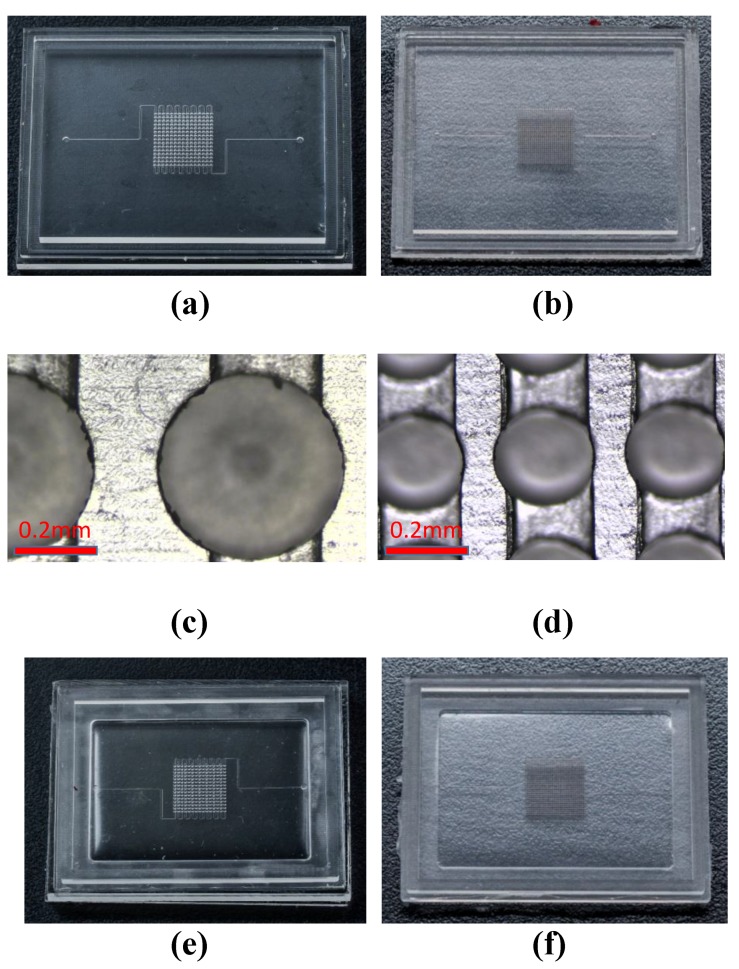
(**a**) Micromilled PMMA mold based on Design I; (**b**) micromilled PMMA mold based on Design II; (**c**) enlarged top-view image of (**a**); (**d**) enlarged top-view image of (**b**); (**e**) PDMS mold cast from (**a**); and, (**f**) PDMS mold cast from (**b**).

PMMA substrates were cut to 50 mm × 37.5 mm × 3 mm and then inscribed using a micromilling machine, which included five major components: a spindle (Nakanishi-E3000c, Nakanishi, Tochigi, Japan), a laser non-contact tool setting system to reset cutting datum following the exchange of milling bit (NC4, Renishaw, Gloucestershire, UK), a numerical controller (M515i, LNC Technology Co. Ltd., Taichung, Taiwan), a compressed air/oil coolant system, and a milling bit holder for tool exchange. Throughout the cutting process, air was supplied to the cutting surface of the PMMA substrate via a nozzle. A two-flute end mill bit (Taiwan Microdrill Co. Ltd., Taichung, Taiwan) was used to manufacture micro cavities on the PMMA substrates. Figure 4a,b, respectively, present the micromilled PMMA molds that are used for Design I and Design II. Figure 4c presents an enlarged image of Figure 4a, whereas Figure 4d presents an enlarged image of Figure 4b, respectively, showing the 15 × 15 and 29 × 29 circular cavity structures that were placed over the microchannels. The microchannel in Design I was 400 μm × 400 μm and the microchannel in Design II was 200 μm × 200 μm.

#### 2.3.2. Cast PDMS Mold

Using the PMMA molds in Figure 4a,b, standard PDMS casting was used to replicate the second mold. PDMS (Sylgard 184, Dow Corning, Michigan, MI, USA) was prepared by mixing pre-polymer and curing agent at a weight ratio of 10:1. After degassing, the PDMS mixture was poured into the PMMA molds, which were then placed in an oven at 80 °C for 1.5 h to undergo curing. The PDMS mold was then removed from the PMMA molds. Figure 4e,f, respectively, present the PDMS molds based on Design I and Design II. When compared to the previous fabrication process, this casting step is an extra step and slightly increased the complication of the whole fabrication process. Carefulness and release agent were used to help smoothly remove the PDMS away from the micromilled PMMA mold since there have many tiny structures in the center of the mold. 

#### 2.3.3. Third Assembly Mold for Fabrication of PDMS Membrane

**Figure 5:**
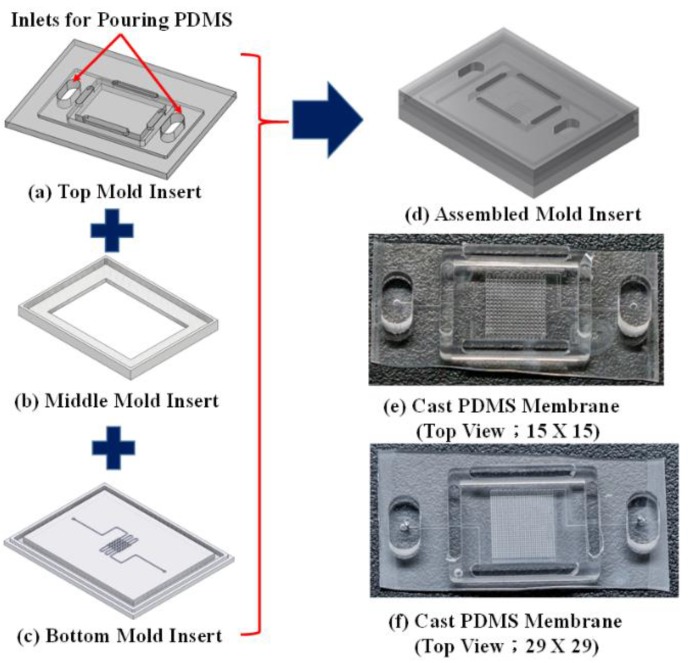
Hybrid mold used to create PDMS membrane: (**a**) top PMMA mold; (**b**) middle PMMA mold; (**c**) bottom PDMS mold shown in Figure 4a,b; (**d**) hybrid assembled mold; (**e**) cast PDMS membrane based on Design I; and, (**f**) cast PDMS membrane based on Design II.

We designed and fabricated a special mold to enable careful control over the height/thickness of the PDMS membrane. Figure 5a–c present the three-piece mold assembly (the detail dimension of these three pieces of molds are shown in Supplementary Material Figure S2), in which the top and middle molds were micromilled PMMA and the bottom was the PDMS mold in Figure 4e,f. The three molds were combined to create the hybrid mold in Figure 5d, which was subsequently used to fabricate the PDMS membrane shown in Figure 3b. After the mold was assembled, it was filled with PDMS and then placed within a vacuum chamber to remove the bubbles that were created during mixing and pouring. The entire mold (with PDMS) was then placed in an oven at 80 °C for 1.5 h to undergo curing. The PDMS membrane was then carefully removed from the assembled mold. Figure 5e,f, respectively, present the PDMS membranes that are based on Design I and Design II.

#### 2.3.4. Using Partial-curing PDMS Bonding for Assembly of PDMS Membrane and Glass Substrate

**Figure 6:**
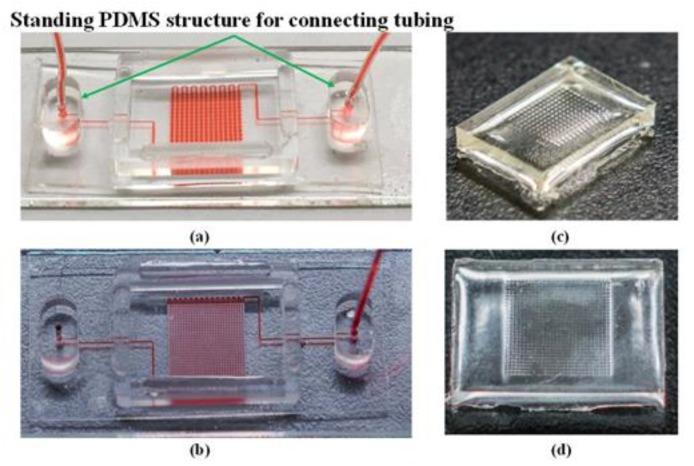
Using partial-curing PDMS bonding to create hybrid microfluidic chip with sufficient bonding strength to allow fabrication of MLAs of high density: (**a**) hybrid chip used for creation of MLAs based on Design I; (**b**) hybrid chip used for creation of MLAs based on Design II; UV adhesive casting applied against hybrid microfluidic chip in Figure 6a,b; (**c**) UV adhesive mold based on Design I; and, (**d**) UV adhesive mold based on Design II.

As described in the last paragraph of the Introduction section, partial-curing PDMS bonding was used to attach the PDMS membrane to the glass substrate. Prior to bonding, the PDMS membrane and glass substrate were cleaned while using IPA, water, and compressed air. Figure 6a,b, respectively, present the bonded hybrid microfluidic chips based on Design I and Design II. The standing structures that are located along the edge of the PDMS membrane were used for the connection of tubing. 

#### 2.3.5. UV Adhesive Casting 

The completed hybrid microfluidic chip was used for UV adhesive casting (Figure 3d). A manual syringe pump was connected to the hybrid microfluidic chip for the injection of red food dye, as shown in Figure 6a,b. Pressure from within the microchannels caused the circular PDMS membrane to deform into a convex shape. UV curable adhesive (Slink P21-A, Slink Adhesive Technology, New Hampshire, NH, USA) was then poured onto the PDMS membrane and then sequentially exposed to UV radiation for 100 s under an irradiation intensity of 50 mW/cm^2^ (Xlite 500, OPAS, Taichung, Taiwan). Following solidification, the UV substrate was demolded from the PDMS membrane for use as a mold in the fabrication of the final PDMS MLAs. Figure 6c,d, respectively, present the cast UV molds based on Design I and Design II.

#### 2.3.6. PDMS Casting of MLAs

**Figure 7:**
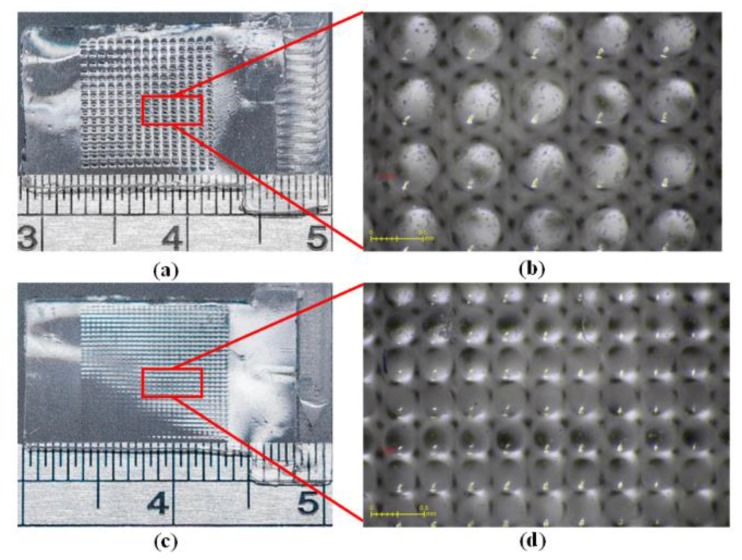
(**a**) Final MLA based on Design I; (**b**) final MLA based on Design II; (**c**) enlarged image showing MLA based on Design I; and, (**d**) enlarged image showing MLA based on Design II.

Once the UV adhesive mold was ready, it was held within a fixture (Figure 3f) to control the thickness of the final PDMS MLAs. Micromilling was used to form cavities in the PMMA substrate, into which was placed the UV molds for subsequent PDMS casting. Again, standard PDMS casting was applied to the PMMA fixture with multiple UV molds for the fabrication of the PDMS MLAs. The overall casting process was performed at 105 °C over a period of three hours. Figure 7a,b, respectively, present regular and enlarged images of the final 15 × 15 MLA. Figure 7c,d, respectively, present regular and enlarged images of the final 29 × 29 MLA.

## 3. Characterization Methods and Results

The efficacy of the revised microfabrication process was assessed in three experiments, designed to estimate the thickness uniformity of the PDMS membrane, the heterogeneous bonding strength that was provided by the partial-curing PDMS bonding technique, and the lens height uniformity of the MLAs.

### 3.1. Uniformity of Membrane Thickness

Ensuring uniform membrane thickness is critical to the performance of MLAs. Figure 5d presents the assembled mold that was used for casting PDMS membrane. In our previous study, we discovered that the thickness of the PDMS membrane determines the pressure that is required to cause deformation and it is closely related to the final lens height of the MLAs. 

Comparisons were made on thickness measurements that were obtained in five locations on each PDMS membrane, as indicated by the red squares in Figure 2. The designed thickness was 75 μm (Design I) and 55 μm (Design II). The experiment results in Table 1 revealed the following information, such as average (Avg.), standard deviation (STD), and coefficient of variance (CV): Design I (average membrane thickness = 80.8 μm/average error = 4.4 μm) and Design II (average membrane thickness = 54.8 μm/average error = 2.8 μm. The CV pertaining to membrane thickness were as follows: Design I (0.04) and Design II (0.07). This is a clear indication that the revised manufacturing technique is capable of producing PDMS membranes of uniform thickness.

### 3.2. Bonding Strength of Partial-curing PDMS Bonding Technique

A proprietary system analysis system was created to measure the burst pressure of the bonded chips. Our primary objective was to assess the efficacy of partial-curing PDMS bonding and to compare the results with those that were obtained using the plasma treatment method in the previous study. Briefly, that method involved exposing the PDMS membrane and the glass substrate to plasma (PDC-32G, Harrick Plasma, New York, NY, USA) in a vacuum chamber for two minutes, before bringing them into contact to produce a hybrid chip. In this experiment, partial-curing PDMS bonding was performed while using the previously described procedure (see final paragraph of the Introduction section).

**Figure 8:**
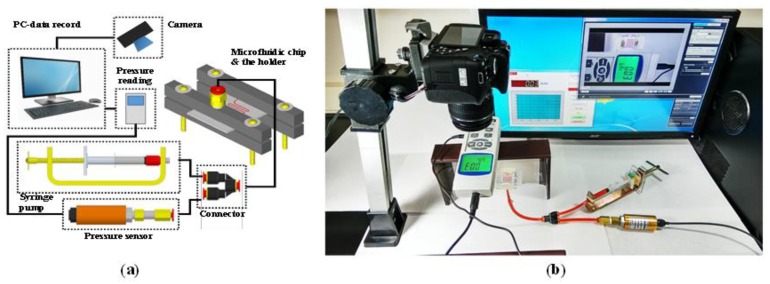
Proprietary system used to perform burst tests to characterize bonding strength: (**a**) schematic diagram of testing system; and, (**b**) actual system used in experiments.

A schematic diagram of the testing system is presented in Figure 8a and the actual system is presented in Figure 8b. The fluidic system included a manual syringe pump for the application of pressure, a pressure sensor (PS100-10 bar, Yalab, Taipei, Taiwan) that was connected to a computer to measure and record experiment data, and an aluminum chip holder to provide tight contact between the microfluidic chip and the tubing during the experiments. A three-way connector was used to ensure the transfer of pressure into the microchannel, while enabling accurate measurements of the accumulated pressure within the microchannel by a sensor. The chip holder ensures that leakage only occurs at the bounded interface between the PDMS and PMMA substrates, i.e., not at the tubing connector. Initially red food dye was injected and completely filled with the microchannel, and then the outlet was blocked in order to accumulate the pressure inside the microchannel when the manual syringe pump was used to increase the pushing pressure. When the leakage was observed, the bonding strength between the PDMS/PMMA chip could then be determined. From the experiments, the maximum bonding strength of the two chips were as follows: plasma-bonded chip (3.5 bars) and partial-cured PDMS = bonded chip (6 bars).

### 3.3. Lens Height Uniformity of MLAs

A third experiment was conducted to determine the lens height of the MLAs. A tool microscope (SL-730, SAGE Vision, Taipei, Taiwan) was focused at the top and bottom of the MLAs, and then the difference between the measured values was used to derive the height of the lens. Measurements were obtained at five locations of the MLAs, as indicated by the red squares in Figure 2. Note that two MLAs of each design were measured, the results of which are listed in Table 2. The coefficient of variance in these measurements was as follows: Design I (0.15) and Design II (0.09). Despite the fact that the PDMS MLA samples were replicated from the same UV mold, the replication process produced slight inconsistencies in terms of lens height.

## 4. Conclusions

This research extends our work in a previous study with the aim of increasing the density of MLAs. This involved modifying two of the fabrication steps. Specifically, we redesigned the first mold and adopted partial-curing PDMS bonding (as an alternative to plasma bonding) to fuse the PDMS membrane to the glass substrate. The revised fabrication method made it possible to create MLAs of far higher density (15 × 15 and 29 × 29), as well as far higher bonding strength. In experiments, partial-curing PDMS bonding achieved a high bonding strength between the PDMS and glass substrate (approximately six bars). The redesign of the mold proved effective in creating PDMS membranes of highly uniform thickness, which is of critical importance to the performance of MLAs. Overall, the revised fabrication method proved to be effective in producing MLAs of high density and high lens height uniformity (variance of 0.1~0.2).

## Figures and Tables

**Table 1:** Uniformity in thickness of PDMS membrane. (Unit: μm)

Design	1st Piece					2nd Piece					Avg.	STD	CV
	1	2	3	4	5	1	2	3	4	5			
Design I (75 μm)	80	82	81	80	81	82	81	80	81	80	80.8	0.79	0.04
Error	5	7	6	5	6	1	2	3	4	5	4.4	1.9	
Design II (55 μm)	55	54	60	52	50	54	50	55	60	58	54.8	3.65	0.07
Error	0	1	5	3	5	1	5	0	5	3	2.8	2.15	

**Table 2:** Uniformity in lens height of MLAs (Unit: μm)

Design	1st Piece					2nd Piece					Avg.	STD	CV
	1	2	3	4	5	1	2	3	4	5			
Design I	94	135	83	92	94	127	107	113	100	110	105.5	16.3	0.15
Design II	25	28	27	32	32	28	31	32	26	30	29.1	2.64	0.09

## Supplementary Materials

The following are available online at https://www.mdpi.com/2072-666X/10/9/572/s1, Figure S1: Overall revised fabrication process: (**a**) micromilling of mold insert; (**b**) casting of PDMS mold from micromilled mold insert; (**c**) casting of PDMS membrane from assembled mold insert, the assembled mold insert is described in Section 2.3.3; (**d**) using partial-curing PDMS bonding to form hybrid microfluidic chip for fabrication of MLAs; (**e**) UV adhesive casting on hybrid microfluidic chip for fabrication of UV adhesive mold insert; (**f**) assembly of UV mold inserts with PMMA fixture to control thickness of final PDMS MLAs; (**g**) completed PDMS MLA; Figure S2: (**a**) The dimension of top micromilled mold; (**b**) The dimension of middle micromilled mold; (**c**) The dimension of the micromilled mold which was used to cast the PDMS membrane. Then the PDMS membrane would assemble with other two molds (a)(b), and be used to create the 3rd mold (Figure 3d).
